# Niclosamide Is a Proton Carrier and Targets Acidic Endosomes with Broad Antiviral Effects

**DOI:** 10.1371/journal.ppat.1002976

**Published:** 2012-10-25

**Authors:** Andreas Jurgeit, Robert McDowell, Stefan Moese, Eric Meldrum, Reto Schwendener, Urs F. Greber

**Affiliations:** 1 Institute of Molecular Life Sciences, University of Zurich, Zurich, Switzerland; 2 3-V Biosciences Inc., Menlo Park, California, United States of America; 3 Institute of Molecular Cancer Research, University of Zurich, Zurich, Switzerland; UCLA, United States of America

## Abstract

Viruses use a limited set of host pathways for infection. These pathways represent *bona fide* antiviral targets with low likelihood of viral resistance. We identified the salicylanilide niclosamide as a broad range antiviral agent targeting acidified endosomes. Niclosamide is approved for human use against helminthic infections, and has anti-neoplastic and antiviral effects. Its mode of action is unknown. Here, we show that niclosamide, which is a weak lipophilic acid inhibited infection with pH-dependent human rhinoviruses (HRV) and influenza virus. Structure-activity studies showed that antiviral efficacy and endolysosomal pH neutralization co-tracked, and acidification of the extracellular medium bypassed the virus entry block. Niclosamide did not affect the vacuolar H^+^-ATPase, but neutralized coated vesicles or synthetic liposomes, indicating a proton carrier mode-of-action independent of any protein target. This report demonstrates that physico-chemical interference with host pathways has broad range antiviral effects, and provides a proof of concept for the development of host-directed antivirals.

## Author Summary

Current antiviral approaches are directed against specific viral targets but are limited in their broad-spectrum potential. Targeting host pathways, on the other hand can have broad antiviral effects, albeit with possible side effects. Here, we report the mode of antiviral action of a small chemical compound, niclosamide. Niclosamide belongs to the salicylanilides, and has been FDA approved to treat parasitic helminthic infestations in humans since decades. In recent years, niclosamide was shown to have anti-neoplastic and antiviral effects. We screened a library of FDA-approved chemicals, and found that niclosamide is an entry inhibitor for a number of pH-dependent respiratory viruses, including influenza virus and human rhinoviruses. Cell biological and biochemical analyses and intracellular pH measurements showed that niclosamide neutralizes acidic membrane-bounded compartments. Niclosamide did not affect the vacuolar ATPase but acted as a protonophore, both in vesicles isolated from cells, and protein-free liposome assays. It blocked rhinovirus infections synergistically with the proton ATPase inhibitor bafilomycin A1. Niclosamide targets endosomes by a mode of action distinct from that of endosomal pH neutralizing agents, such as chloroquine, which accumulates in acidic endosomes.

## Introduction

The discovery of antibiotics against microbes in the early 20^th^ century has had a major impact on society and saved countless lives [1]. Yet, broad range inhibitors against viral pathogens are presently not available. Classical antiviral strategies have targeted viral structural or enzymatic motifs with high specificity and potentially low side effects. These agents are, however, prone to viral evasion, and select for resistant strains [2]. To identify novel chemical inhibitors of virus infection we performed a high-content, image-based infection screen using a library of approximately 1200 known bioactive and food and drug administration (FDA) approved small compounds for their ability to inhibit human rhinovirus (HRV) 16 infection of HeLa cells.

HRV belong to the genus *Enteroviridae* of the picornavirus family. Picornaviruses are small, non-enveloped viruses with a capsid of 28–30 nm in diameter and a plus-sense, 6500–9000 nucleotide RNA genome. The genome encodes a single poly-protein, which is proteolytically processed during infection [3]. HRVs comprise more than one hundred known serotypes. They are the most frequent viral infections among humans and the predominant cause of the common cold [4]. HRV infections can pose severe health risks in patients with pre-existing airway conditions, including chronic obstructive pulmonary disease, asthma or cystic fibrosis. This may relate to the observation that HRVs are not limited to the upper respiratory tract but also infect the lower respiratory tracts [4]. Two receptor groups have been defined for the species A and B viruses. The minor group viruses comprise twelve members utilizing low-density lipoprotein receptor (LDLR) proteins for cell entry, and the major group uses the intracellular adhesion molecule 1 (ICAM1) [5]. These infections are typically pH-dependent and require the passage of viruses through acidic endosomal compartments. The recently discovered species C HRVs, which are prevalent in young children appear to use distinct but unknown attachment sites for infection [6].

There are no vaccines or effective antivirals available against HRV, despite the fact that non-influenza related viral respiratory infections, predominantly by HRV, lead to an economic loss of USD 40 billion per year in the US alone and are among the top reasons for prescription of antibiotics [7]. Earlier developments of small compounds against HRV had targeted capsid or viral protease functions, for example pleconaril or rupintrivir [8], [9]. None of these antiviral molecules passed phase III clinical trials, or was approved by the FDA for broad human applications.

We identified niclosamide (5-chloro-N-(2-chloro-4-nitrophenyl)-2-hydroxybenzamide) as an inhibitor of HRV16 infection. Niclosamide is an FDA approved anti-helmintic compound used in humans since more than forty years [10]. It has been applied as an agrochemical and in human therapy. It is well tolerated by rats with an acute oral toxicity LD_50_ dose larger than 5 g/kg body weight and only a marginal decrease in haemoglobin concentration and erythrocyte count occurred when male and female rats were given niclosamide at 5 g/kg/day for four weeks [11]. Niclosamide was originally developed at *Farbenfabriken Bayer* based on the observation that bithionol (2,2′-sulfanediyl-bis(4,6-dichlorophenol)) had potent antihelmintic activity. Later work on hydroxyl-biphenyls led to the discovery of niclofolan (5,5′-dichloro-2,2′-dihydroxy-3,3′-dinitrobiphenyl), which was modified to niclosamide. Niclosamide was introduced into the market in 1960 under the trade name *Bayluscide (Bayer 73),* and has been used over decades to treat gastrointestinal tapeworm infections, both in humans and animals, [for an overview, see 12]. Niclosamide is receiving renewed attention due to antiviral effects against severe acute respiratory syndrome (SARS) virus [13], anti-anthrax toxin properties [14], and anti-neoplastic activity [15]. In addition, niclosamide is a potent inducer of LC3-positive autophagosomes [16], an inhibitor of the Wnt/Frizzled pathway [17], a suppressor of the autonomous notch-signalling pathway [18], and an inhibitor of mTOR signalling [19]. In addition, it uncouples mitochondrial oxidative phosphorylation [20], thus slowing down cell growth.

## Results

### Niclosamide is a broad range antiviral against pH dependent viruses

**Figure 1:**
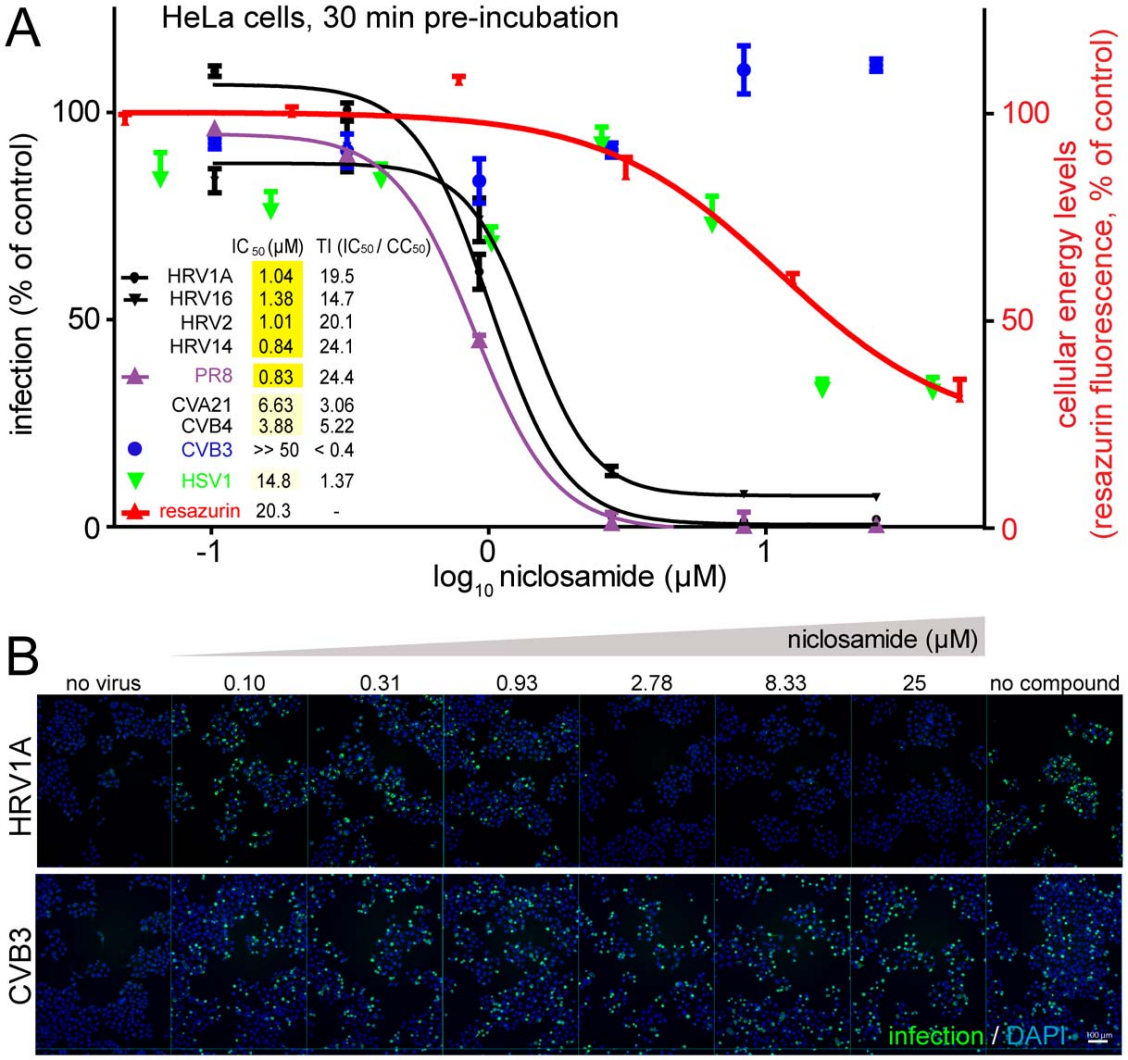
Niclosamide is a dose-dependent inhibitor of enterovirus and influenza virus infections. A). Niclosamide protects HeLa cells from HRV1A, 2, 14 and 16 infections (n = 2), and A549 cells from Influenza A/PR8 (n = 2), while CVB3 (n = 2) and HSV1 (n = 6) infections were not significantly affected, compared to the toxicity effects determined by resazurin measurements. Mean and SEM values are shown, percent relative to DMSO-treated control cells (100%). The inset shows the IC_50_ concentrations for half maximal infection inhibitions and the therapeutic indices (TI), defined as the ratio of IC_50_/CC_50_, where CC_50_ is the concentration at 50% cell viability. B). Sample images showing the effect of niclosamide on the formation of dsRNA replication centers (mABJ2, Alexa488, green) at 7 h pi. Nuclei are stained with DAPI (blue). Bar = 100 µm.

In an image-based high-content infection screen of HeLa cells, we identified niclosamide from a library of 1200 known bioactive compounds as a potent, low micromolar inhibitor of HRV16 infection. We tested the efficacy of niclosamide to block picornavirus infections using a panel of different enteroviruses, comprising four HRVs and coxsackieviruses (CV). We found that pH-dependent HRVs using the LDLR family (HRV1A, 2), the ICAM1 tropic major group (HRV14, 16) and the genetic groups A (HRV1A, 2 16) and B (HRV14) were sensitive to niclosamide at IC_50_ (the concentration with 50% of the maximal inhibition) of 0.84 to 1.4 µM (Figure 1A, B and Figure S1A in Text S1). The largely pH-independent coxsackie adenovirus receptor (CAR)-tropic CVB3 and CVB4 [21], and the ICAM1-tropic CVA21 were broadly unaffected at 1 µM and had IC_50_ values of >50 µM, 3.88 µM and 6.63 µM, respectively. This is also reflected in the therapeutic index (TI), reflecting the ratio of IC_50_ to CC_50_, the latter being the concentration with half maximal toxicity. We measured cellular energy levels using a resazurin fluorometric assay to assess the toxicity of niclosamide. Toxicity was clearly separated from antiviral efficacy against HRV1A, 2, 14, and 16 at least 10-fold (see Figure 1A and Figure S1B in Text S1). The toxicity profile closely matched the antiviral efficacy against herpes simplex virus (HSV1), which had IC_50_ values exceeding 10 µM.

In contrast, niclosamide effectively inhibited influenza virus (strain A/Puerto Rico/8/34/H1N1 depicted *PR8*) with an IC_50_ of 0.83 µM in human lung adenocarcinoma epithelial A549 cells, a cell line fully permissive for influenza virus replication (Figure 1A). Niclosamide was also an effective antiviral in non-transformed cells, including WI-38 human embryonic diploid airway cells infected with HRV1A (IC_50_ 1.3 µM, Supplementary Figure S1C in Text S1). However, it did not affect the inhibition profile when viruses were pre-treated with niclosamide at up to 400 µM at 37°C for 30 min, suggesting a non-virus target (Figure S2 in Text S1). The cellular inhibition profile of niclosamide closely resembled that of bafilomycin A1 (BafA1), which strongly inhibited HRV1A, 2, 14, 16 and mildly CVB4 but not CVB3 (Figure S3 in Text S1). The profile was also similar to ammonium chloride addition experiments (see Figure S7B in Text S1, and data not shown).

The high mutation rate and *quasi-species* nature of picornaviruses facilitate the emergence and selection of drug-resistant escape mutants, particularly if the restriction is directed against viral factors [22]. We addressed this issue by *blind* propagation of HRV1A and CVB3 in the presence of niclosamide at effective concentrations ranging from 0.12–10 µM in approximately 24 HRV replication cycles. We did not observe a shift in sensitivity of propagated HRV1A to niclosamide (Figure S4 in Text S1). It is not known if this was due to low genetic complexity of the inoculum, or lack of spontaneous mutations during passaging. Collectively, these data show that pH-dependent infections are more affected by niclosamide than pH-independent infections.

### Niclosamide inhibits HRV entry

**Figure 2:**
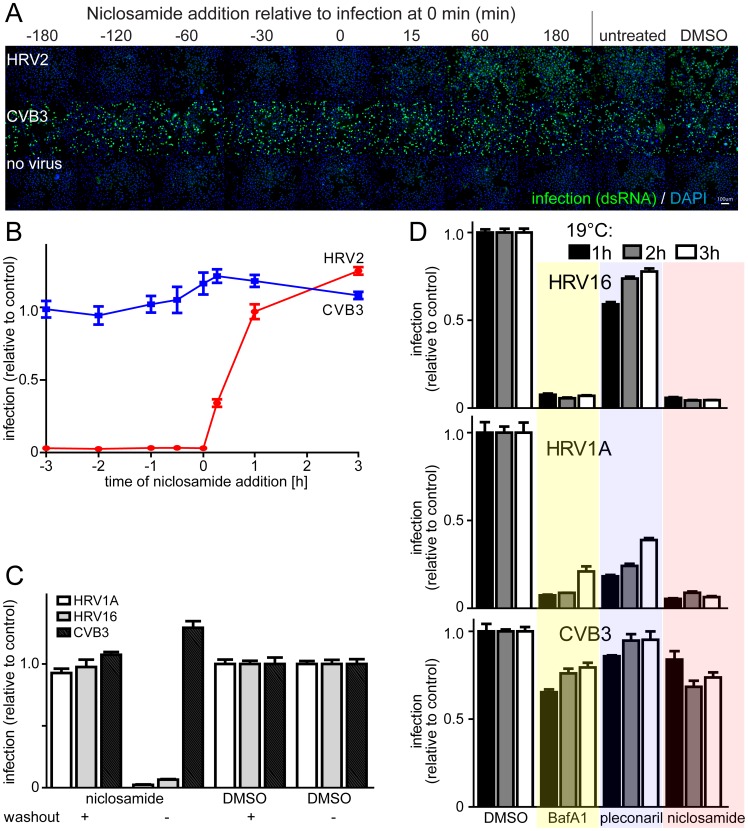
Niclosamide inhibits virus entry post the 19°C compartment. A). Time course of niclosamide (12.5 µM) addition and assessment of HRV2 infection of HeLa cells, including DAPI staining for nuclei. B). Quantification of HRV2 and CVB3 infections from panel A with means of infection values including SEM (n = 4). C). Impact of a drug washout on niclosamide efficacy against HRV1A, 16 and CVB3. Cells were pre-incubated with 10 µM of compound for 30 min and either washed three times with PBS or drug. Mean values and SEM of n = 4 are shown relative to DMSO treated control cells. D). Efficacy of BafA1 (50 nM), pleconaril (0.5 µg/ml) and niclosamide (5 µM) added to cells, which had been inoculated with HRV16, 1A or CVB3 for 1, 2 or 3 h at 19°C. Mean infection values and SEM of n = 3 are shown relative to DMSO controls.

To assess at what stage of the viral life cycle niclosamide acted, we added the compound before or post infection (pi) of HeLa cells with HRV or CVB3 (Figure 2A, B, Figure S5A in Text S1). The results show that addition of niclosamide before the genome release was necessary for maximal inhibition of HRV infections, with half maximal effects before or at 30 min pi, while CVB3 infection was unaffected. This suggested that niclosamide inhibited an early step of infection, presumably virus entry. These results were similar to time-course experiments with the entry inhibitor pleconaril [21], which binds to HRV capsids, or with BafA1, which neutralizes acidic endosomes by blocking the vacuolar ATPase (see Figure S3 in Text S1).

To test if niclosamide was an irreversible inhibitor we pre-treated cells with the compound for either 30 min or 18 h. Washing the cells with drug-free medium completely abolished the antiviral effect in both cases (Figure 2C and Figures S5B and S5C in Text S1). This suggested that the mode of inhibition did not involve high affinity drug targets or irreversible metabolic changes in the cells even when exceeding 10-fold IC_50_ and long-term incubation. To better define the inhibition mode of niclosamide, we performed 19°C trapping experiments. At this temperature, uptake of cargo into early endosomes is allowed, but membrane maturation and sorting from these early compartments are strongly slowed down [23]. HRV1A or HRV16 was internalized at 19°C for 1, 2 or 3 h followed by treatment with niclosamide at 37°C, or with drugs affecting capsid conversion. Here, we used the term ‘capsid conversion’ to measure cellular cues priming the virus for RNA release [5], for example virus-receptor interactions or low endosomal pH. Capsid conversion was measured by the sensitivity of HRV to pleconaril, which binds to capsid and interferes with immediate early receptor binding steps of ICAM1-tropic HRVs [24], and with endosomal conversion of (v)LDLR- tropic HRVs, [unpublished data, and 25]. Our data show that the ICAM1-tropic HRV16 readily bypassed the pleconaril block at 19°C (Figure 2D). The (v)LDLR-tropic HRV1A, in contrast, remained at least partly pleconaril-sensitive, indicating that it did not get past the 19°C step towards an acidic compartment where the pH-dependent conversion step occurs, as shown earlier for the (v)LDLR-tropic HRV2 [26]. As expected, BafA1 inhibited the infection with both HRV1A and HRV16, but not the pH-independent CVB3, when added after 1, 2 or 3 h at 19°C. Likewise, niclosamide blocked HRV1A and HRV16 but not CVB3 when added after the 19°C block. We conclude that niclosamide inhibited HRV1A and HRV16 infections after receptor binding and transport of virus to early endosomes.

### Niclosamide neutralizes pH and alters the distribution of endosomes

**Figure 3:**
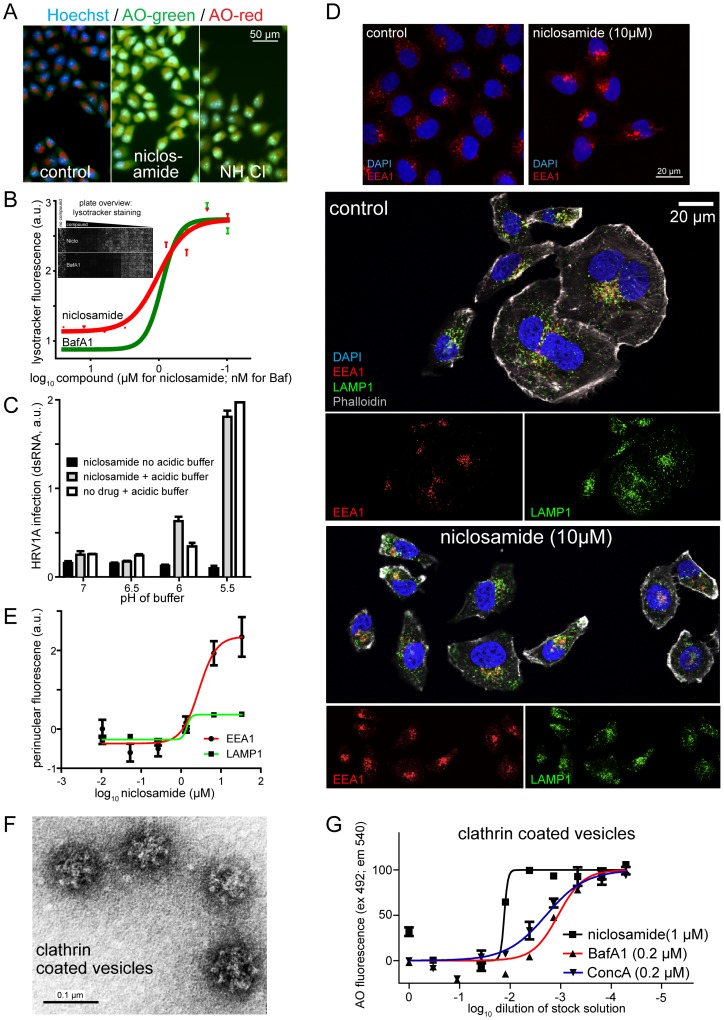
Niclosamide neutralizes acidic endosomal pH and alters the distribution of endosomes. A). Ratiometric live cell imaging of acridine orange (AO) green and red fluorescence. Nuclei were stained with Hoechst. B). The mean and SEM values of lysotracker fluorescence from single cell measurements are plotted against niclosamide (µM) and BafA1 (nM) (n = 4) with an overview of lysotracker fluorescence from a 96-well plate. C). Low extracellular pH bypasses niclosamide's antiviral effect. Mean and SEM values of dsRNA-positive cells (n = 2). D). Influence of niclosamide on the distribution of EEA1 (red) or LAMP1 (green) positive endosomes observed in single section confocal micrographs where nuclei (blue) were stained with DAPI and filamentous actin (grey) with phalloidine. E). Quantification of the perinuclear intensity of EEA1 and LAMP1 positive endosomes of cells from automated microscopy and single cell analyses relative to wild type levels (0) with means and SEM (n = 8). F). Electron micrograph of a negatively stained cow brain CCV preparation. G). Percent normalized fluorescence of acridine orange (AO) (excitation 492 nm, emission 540 nm) of control and inhibitor-treated CCV. The 100% signal is equivalent to the DMSO control treated vesicles.

The majority of niclosamide-sensitive viruses in this study, namely, HRVs 1A, 2, 14, 16, and influenza virus PR8 require a low-pH step for infectious entry [24], [27], [28]. We therefore tested if niclosamide neutralized the low endosomal pH, as measured by ratiometric imaging of acridine orange (AO) fluorescence [29] (Figure 3A). AO accumulates in vesicular compartments and emits red fluorescence, supposedly due to quenching in acidic compartments. Upon treating cells with the lysosomotropic agent NH_4_Cl, or the v-ATPase inhibitor BafA1 the emitted fluorescence of AO shifted from red to green, indicating de-quenching upon endosomal pH neutralization. Ratiometric live cell measurements showed that niclosamide increased the vesicular pH, similar to NH_4_Cl or BafA1 (Figure 3G, and Figure S6A and B in Text S1). These results were confirmed by lysotracker DND99 measurements, which showed a steep decline in fluorescence upon treatment with micromolar concentrations of niclosamide, or nanomolar concentrations of BafA1 (Figure 3B). This indicated that niclosamide neutralized low pH endosomal compartments.

It is well established that endolysosomal acidification is largely driven by v-ATPases by consumption of ATP, [reviewed in 30]. To test if niclosamide affected the v-ATPase activity, we isolated bovine brain clathrin-coated vesicles (CCV), and measured the hydrolysis of ATP by the production of inorganic phosphate (P_i_). While niclosamide had no effects on ATP consumption, the v-ATPase inhibitors BafA1 or concanamycin A (Conc A) completely blocked the production of P_i_ (Figure S6C in Text S1, for an electron micrograph of isolated CCV, see Figure 3F). This indicated that niclosamide did not inhibit the v-ATPase activity.

We next tested if the antiviral effect of niclosamide was due to blocking of endosomal acidification. For this, we trapped HRV1A in the 19°C compartment for 2 h in presence of niclosamide and incubated the cells in medium with low pH for 1 h, followed by normal infection medium without niclosamide for 6 h in presence of pleconaril to inactivate non-converted particles. We found that the addition of pH 5.5 or 6 medium reversed the inhibitory effect of niclosamide on 19°C trapped viruses and promoted infection (Figure 3C). The infection stimulation was more pronounced at pH 5.5 than 6, possibly due to activation of virus penetration through the plasma membrane. Similar results were obtained with BafA1 treatment of 19°C trapped virus, where the inhibition was reversed by pH 5.5 or 6, albeit pH 5.5 was more efficient than pH 6 (data not shown). Notably, the treatment of cells with pH 5.5 buffer, which leads to acidification of the cytosol [31] did not inhibit HRV1A infection. Together, the results indicate that niclosamide affected a low pH-dependent step of the entry process at or past the 19°C compartment.

Early endosomes are an important passageway for a variety of viruses and have a mildly acidic pH of 6 to 6.5 [32]. Since we observed that niclosamide inhibited viruses, which were strongly pH dependent and also less pH dependent, we investigated if niclosamide affected the phenotypes of early endosomes by staining for early endosomal antigen 1 (EEA1). We found morphologic alterations of EEA1 positive structures in niclosamide-treated cells, specifically endosomal clustering in the perinuclear area (Figure 3D, E). The perinuclear intensity of LAMP1 (lysosomal associated membrane protein 1) positive structures was less affected. Notably, BafA1 and to lesser extent the lysosomotropic agents NH_4_Cl and chloroquine had comparable phenotypic effects as niclosamide (Figure S6D and E in Text S1). Similar results were also obtained with normal diploid WI-38 cells indicating cell type independent effects (data not shown). To test if niclosamide directly affected the pH within endosomes, we measured the pH of isolated CCV by the AO red fluorescence assay. Niclosamide, BafA1 and ConcA neutralized the pH of CCV in a dose-dependent manner (Figure 3F, G), notably with lower IC_50_ compared to cellular assays, as seen frequently in cell free assays. Collectively, the data indicate that niclosamide neutralized the pH of acidic endosomes.

### Antiviral efficacy of niclosamide co-tracks with neutralization of endosomal pH

**Figure 4:**
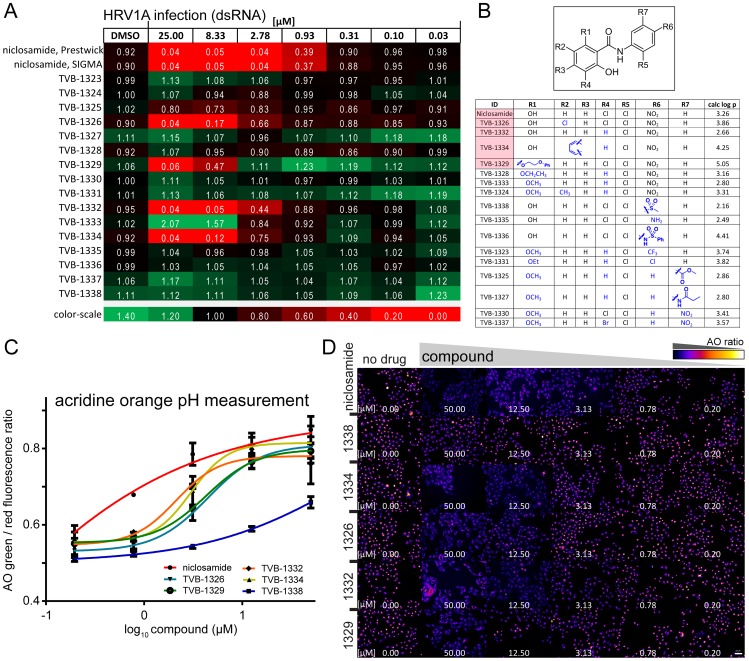
Antiviral efficacy of niclosamide related compounds co-tracks with endo-lysosomal neutralization. A). Efficacy of niclosamide and sixteen structurally related compounds against HRV1A infection of HeLa cells. Infection relative to DMSO treated controls (value 1) and means are indicated (n = 3). Infections and analyses as shown in Figure 1. B). Chemical structures of the sixteen niclosamide-related compounds dubbed as TVB compounds. C). Acridine orange (AO) assay for pH-neutralization of endosomal compartments of five TVB compounds with antiviral efficacy. The perinuclear intensity of the red and green AO fluorescence was quantified. Mean and SEM values are shown (n = 2), representing more than 500 cells per condition. D). Overview of green/red ratiometric fluorescence of cells treated with increasing concentrations of indicated compounds. False color images correspond to the data in (c).

Niclosamide is a weak acid with an estimated pKa of 5.6 [33]. At pH 7.4, about 99% of the molecules are in the deprotonated form. The octanol/water distribution coefficient (log P) of niclosamide increases from pH 7.4 to 6 [33]. To test if there was a correlation of antiviral activity and neutralization of endosomal pH, we performed a structure-activity relationship study with a library of sixteen niclosamide-related compounds, each carrying different functional groups. As a positive control we included niclosamide from two different sources, Prestwick and SIGMA-Aldrich, and found that both reagents had a virtually identical efficacy against HRV1A (Figure 4A). Four of the sixteen niclosamide-related compounds had antiviral activity, comparable to niclosamide (Figure 4A and 4B). TVB-1326, 1332 and 1334 all contained features that stabilized their protonated forms, which suggested that the hydroxyl group at R1 or the central amide NH were the pH sensitive group. Condensation of the R1 hydroxyl group with ethoxy-phenyl (TVB-1329) did not diminish the antiviral efficacy compared to niclosamide, indicating that the R1 hydroxyl group alone was not essential. However, substituting the R1 hydroxyl proton with an alkyl group and replacing the electro-negative R4 Cl group with hydrogen (TVB-1328, 1331, 1333, and 1324) eliminated antiviral activity. The same was true when the electron-withdrawing nitro group at R6 was replaced by less electron-withdrawing groups (TVB-1338, 1335, 1336). The importance of the electro-negative groups on the benzene rings was confirmed by the reduced antiviral efficacy of TVB-1323, 1325, 1327, 1330 and 1337.

We confirmed the antiviral activity of TVB-1326, 1332, 1334 and 1329, but not of the control compound TVB-1338 against the pH-dependent HRV1A, 2, 14, 16 and influenza virus PR8 and to a lesser extent CVB4 and CVA21 (Figure 4A, and Figure S7A in Text S1). Similar to niclosamide, the antiviral active compounds inhibited infection with HRV1A or HRV16 when added during the early phase (30 min) but not when added post entry at 120 min (Figure S7B in Text S1). CVB3 was only weakly inhibited by the treatment with niclosamide or the compounds TVB-1326, 1332, 1334 and 1329. The inhibition profiles were comparable with the entry inhibitors BafA1 and NH_4_Cl, although NH_4_Cl slightly affected the infection with CVB3. The latter effect is likely due to pleiotropic effects of NH_4_Cl on cell physiology. Furthermore, TVB-1326, 1329, 1332, 1334 but not TVB-1338 readily neutralized acidic endosomes as determined by the ratiometric AO assay or lysotracker fluorescence analyses in HeLa cells (Figure 4C, D, and Figure S7C in Text S1). Collectively, our studies indicated that the inhibition profile of niclosamide and related compounds against acid-dependent viruses closely matched with the pH neutralization profile of the compounds.

### Niclosamide neutralizes protein-free acidic liposomes and blocks infection in synergy with BafA1

**Figure 5:**
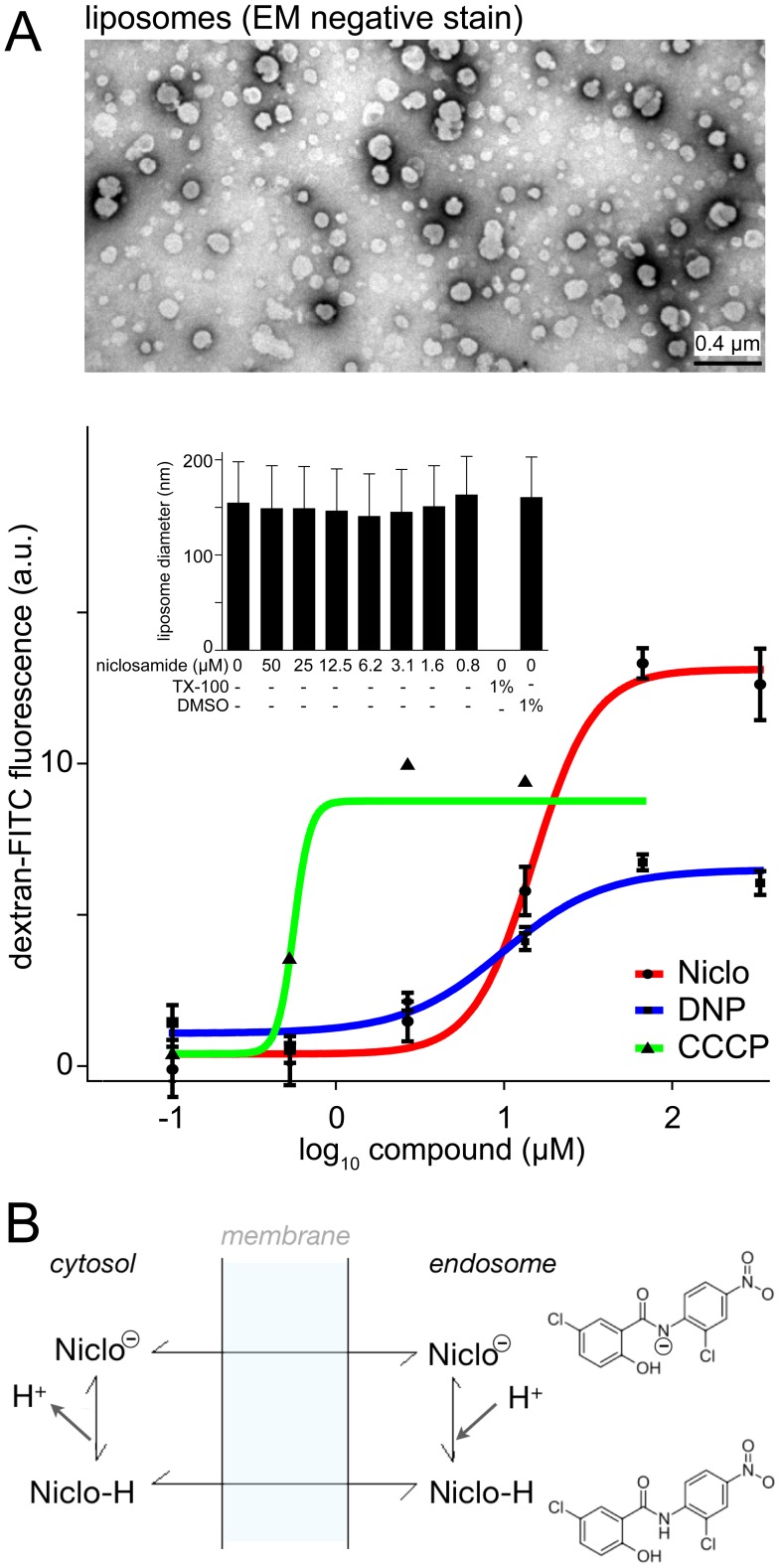
Niclosamide neutralizes acidic liposomal pH similar as the protein gradient uncoupling agents DNP or CCCP. A). 200 nm sized liposomes were prepared in presence of dextran-FITC (1 mM, 4.4 kDa) at pH 5.15 (top panel), which quenches FITC-fluorescence and dequenched by the protonophoric action of niclosamide, 2,4-DNP (2,4-di-nitrophenol) or m-CCCP (m-chlorophenylhydrazone). The integrity of liposomes upon treatment is shown as mean vesicle size (insert bar-graph). B). Model of the protonophore mode of action of niclosamide. Niclosamide readily passes through biological membranes due to its lipophilic nature, as indicated by log P = 4.48 at pH 7.0 [33]. Niclosamide (pKa of 5.6) is protonated in the acidic compartment of endosomes, and thereby increases its lipophilicity (log P = 5.63 at pH 5.7). This may enhance its partitioning into the lipid bilayer. In the pH-neutral cytosol a proton dissociates and the compound can interact again with membranes to repeat the cycle. Note that the location of the proposed negative charge on niclosamide is not known.

To further explore the mode of action of niclosamide, we tested the protonophoric ability of niclosamide in protein-free unilamellar liposomes loaded with dextran-FITC. To quench FITC fluorescence the liposomes were loaded at pH 5.15 in a manner similar to endosomes loaded with dextran-FITC [34]. Upon short incubation with niclosamide the FITC-fluorescence was rapidly dequenched with half maximal efficacy at 14 µM (Figure 5A). Fluorescence dequenching was similar to the protonophores DNP (dinitrophenol) or CCCP (carbonylcyanid-m-chlorophenyl-hydrazone), validating the assay system. Notably, niclosamide did not affect the integrity of the liposomes, indicated by laser scatter size determination and electron microscopy of negative stained liposomes, whereas the detergent Triton X-100 disintegrated the liposomes. These data supplied direct evidence that niclosamide acted as a proton carrier from acidic vesicular lumen to the pH neutral cytosol (Figure 5B).

**Figure 6:**
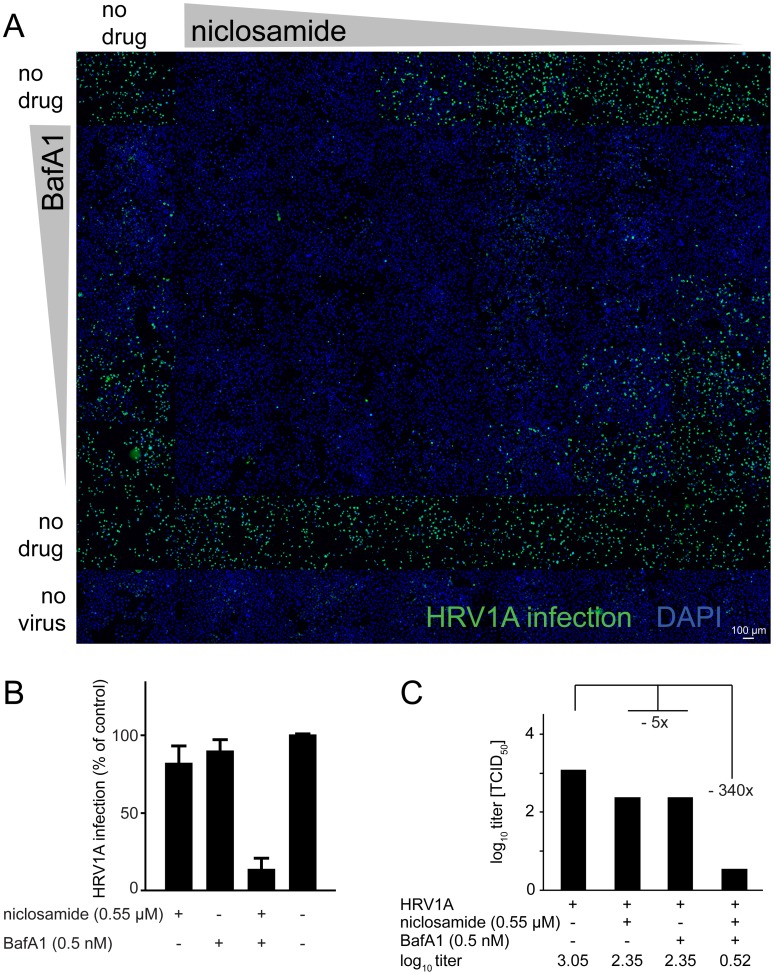
Synergistic antiviral efficacy of niclosamide and the v-ATPase inhibitor BafA1. Single round infections (a, b) with an overview fluorescence microscopy montage of HeLa cells infected with HRV1A (green) in presence of increasing concentrations of niclosamide, BafA1 or combinations thereof with cell nuclei stained with DAPI (blue). Bar is 100 µm. Viral titer production is indicated in panel (C).

We next tested if combination treatments of cells with niclosamide and BafA1 had additive or synergistic antiviral effects. Pre-incubation of cells with serial dilutions of niclosamide combined with BafA1 showed a strong “additive” inhibition of HRV1A infection, as determined by single cell, high-content infection assays (Figure 6A, B). At low concentrations of niclosamide and BafA1 synergistic inhibition of infection was found as determined with MacSynergy II (Figure S8 in Text S1). The synergistic effects were in strong agreement with results from full cycle assays where low concentrations of niclosamide (0.55 M) or BafA1 (0.5 nM) alone decreased virus titers about five-fold, and with the drug combination >300-fold (Figure 6C). Together, the data indicate that host interference at the level of endosome acidification by two distinct mechanisms synergistically blocks virus entry and infection.

## Discussion

To identify tools against human rhinoviruses, we screened a library of FDA-approved chemical compounds [35]. Among the top hits against HRV16 infection was niclosamide. Niclosamide is well tolerated in humans. It can be orally used at 2 g per adult for cestocidal treatment, leading to maximal serum concentrations of 0.25 to 6.0 µg/ml corresponding to 0.76–18.35 µM, which is well within the anti-viral active concentration range and is non-toxic in humans [36]. Niclosamide is a salicylamide compound, similar to the thiazolide nitazoxanide, a pro-drug of the active compound tizoxanide [37]. Nitazoxanide was shown to have a broad spectrum of efficacy, albeit with an unknown mode of action. It has anti-protozoan activity and efficacy against influenza virus, hepatitis B, rotavirus, norovirus and is in phase II clinical trials for chronic hepatitis in combination therapy with interferon [38]–[40]. It was also reported to inhibit the maturation of influenza virus hemagglutinin, presumably in the Golgi apparatus and to disrupt the membrane potential of *Mycobacterium tuberculosis* [41], [42]. Nitazoxanide (pKa 5.8) also has antiviral efficacy against hepatitis C virus (HCV), presumably by activating protein kinase R (PKR) [PKR, 43]. However, using PKR-knock out mouse embryo fibroblasts [44], we did not find a role of PKR in niclosamide mediated block of infection with HRV1A (data not shown). Nonetheless, niclosamide inhibits infection with the pH-dependent viruses, HCV and SARS coronavirus [40], [45], [46], but the mechanism of inhibition was unknown.

Here, we show that niclosamide inhibits infection with human rhinoviruses and influenza virus by blocking the acidification of the endolysosomal compartments. Niclosamide did not affect viral uptake into cells, since it acted post conversion of HRV upon receptor engagement and post the 19°C endocytic compartment. Instead, niclosamide acted as a proton carrier and synergized with the inhibitory effects of the v-ATPase inhibitor BafA1. This process affects pH homeostasis of endosomes, which is tightly maintained in normal cells, [reviewed in 47]. The mode of action of niclosamide is different from the v-ATPase inhibitor BafA1, or the lysosomotropic agents ammonium chloride (NH_4_Cl) or chloroquine. The latter are trapped as cationic species in the acidic organelles owing to their basic pKa, thereby elevating the endosomal pH [48]. We speculate that the lower apparent toxicity of niclosamide compared to v-ATPase inhibitors could be due to a pH-tuned action based on the mildly acidic pKa of niclosamide. This would be different from bafilomycin, which blocks the rotation of v-ATPase subunits during proton flux independent of the luminal pH [30]. Niclosamide did not affect the ATP hydrolysis in purified CCV unlike BafA1 and required lower micromolar concentrations for antiviral efficacy than lysosomotropic agents, while NH_4_Cl is effective at low millimolar concentrations [49], [50]. Comparably, chloroquine has an IC_90_ of 20 µM for hepatitis C virus [51], or mono-dansyl-cadaverine has an IC_50_ of 50–100 µM against dengue virus [52]. Unlike NH_4_Cl, niclosamide does not apparently induce intracellular vacuolization, suggesting that it does not accumulate in the aqueous lumen of low pH compartments. This is supported by the observation that the hydrophobicity index of niclosamide increases from neutral to acidic pH as shown by experimental data [33].

Niclosamide acts differently than classical ionophores. Nigeracin, for example, is a mitochondrial proton gradient uncoupler at high concentrations of potassium ions (K^+^), and thereby catalyzes the electro-neutral exchange of K^+^ for protons without affecting the membrane potential [53]. The infection block by niclosamide was reversed by addition of low pH medium, suggesting that the endosomal pH rather than a pH gradient across the endosomal membrane is crucial for HRV infection. This is distinct from valinomycin, which disrupts the membrane potential by shuttling Na^+^ and protons. It is also unlikely that niclosamide neutralizes the endosomal pH by inhibition of counter-ion influx, since cation efflux rather than anion influx accompanies endosomal acidification at least in the mildly acidic range [54]. Further, the chemical properties of niclosamide make it unlikely that it acts as an alkali ionophore, such as monensin, which forms zwitter-ionic complexes with monovalent cations and transports them through membranes [55]. Interestingly, niclosamide and its related compounds not only inhibited the pH-dependent viruses HRV1A, 2, 14, and 16, but also, albeit moderately, the less pH-dependent strain CVB4. These results are in good agreement with the infection inhibition profile of EIPA (ethyl-isopropyl-amiloride), an inhibitor of the sodium-proton exchanger regulating endosomal pH [56]. For example, the niclosamide-sensitive CVB4 was as sensitive to EIPA as HRV1A, 2, 14 or 16, while the niclosamide-insensitive CVB3 was not affected by EIPA (data not shown). This suggests that the ionic milieu of endosomes affects infections, possibly by controlling membrane trafficking or homeostasis. This was supported by the observation that niclosamide altered the mildly acidic EEA1-positive early endosomal compartments. It is possible that luminal pH controls the interaction of the membrane trafficking regulator Arf6 with the c-subunit of the v-ATPase complex, and the Arf GTP-exchange factor ARNO with the membrane embedded a2-subunit, which anchors the ATPase complex in the membrane and controls proton transport [57].

By showing that niclosamide induces the neutralization of endosomal pH we offer an explanation for a variety of cell biological effects attributed to this compound in the past. Niclosamide has been safely used in humans since decades after optimizations for the treatment of gastrointestinal parasites. It can now also be tested in animal models for viral infection inhibition, ideally by application via the inhaled route in order to obtain high local concentrations for maximal efficacy. Modifications in its chemical structure, e.g. generation of a pro-drug may prove useful to advance the potential of physico-chemical interference for the treatment of respiratory viral infections. The fact that niclosamide is a protonophore independent of protein targets with broad-spectrum antiviral efficacy enforces the concept of host targeting for antiviral therapies. It may spur the development of novel directed protonophores tuned to particular cellular compartments.

## Materials and Methods

### Viruses and cells

Viruses and cells where essentially prepared as described [21]. HeLa cervical carcinoma cells strain Ohio (from L. Kaiser, University Hospital Geneva, Switzerland), primary human embryonic lung Wi-38 cells (American Type Culture Collection) and human lung adenocarcinoma epithelial cells A549 (both from ATCC) were cultured in Dulbecco's Modified Eagle Medium (DMEM) supplemented with L-glutamine, non essential amino acids and 10% fetal bovine serum (all Sigma) at 37°C and 5% CO_2_ in a humidified incubator and propagated sub-confluent twice a week. In all experiments passage numbers were kept at a maximum of 25 post thawing. For infection experiments in 96 well plates (Matrix, Thermo Scientific) 14000 cells were split in 100 µl the day before the experiment. HRV serotypes 1A and 16 were from W.M. Lee (University of Wisconsin, Madison, WI, USA), HRV 2 and 14 from L. Kaiser (University Hospital Geneva, Switzerland) and CVB3, B4 and A21 from T. Hyypiä (University of Turku, Finland). Influenza strain A (Puerto Rico/8/34/H1N1) grown in MDCK cells was a kind gift from J. Pavlovic (University of Zürich, Switzerland). HSV-1 expressing GFP from the major CMV promoter was a kind gift from C. Fraefel (University of Zürich). The genetic nature of HRV serotypes used was verified by diagnostic sequencing [21].

### Chemicals

BafA1 and ConcA were from Sigma-Aldrich & Enzo Life Sciences (Switzerland). Niclosamide was obtained from two sources, Prestwick (Illkirch, France) and Sigma. The batch from Sigma was 100% pure by thin layer chromatography (Batch 058K1597, see supplier homepage for certificate of analysis). All other TVB-compounds were supplied by 3-V Biosciences (Menlo Park, CA, USA). The original screening library of FDA approved compounds was from Prestwick.

### Infection assays and automated high throughput infection analysis

A single cell high-content, image based infection assay was applied to measure infection in high throughput format [21]. Viruses were added to cells in infection medium containing BSA (DMEM containing 25 mM HEPES if used outside of a CO_2_ cell culture incubator, supplemented with L-glutamine, 30 mM MgCl_2_ and 0.2% BSA (Sigma) for CV and HRV), or in infection medium containing trypsin-TPCK (DMEM supplemented with L-glutamine and TPCK-trypsin 1 µg/ml (Sigma) for FLU/PR8) and normal growth media for HSV. For all experiments, the multiplicity of infection (moi) was chosen such that approximately 20–40% of the cells were infected at 7 h post infection (pi) (HRV &CV), 8 h pi (FLU) or 18 h pi (HSV). Cells were fixed by adding 1/3 volume of a 16% para-formaldehyde solution, washed with PBS, PBS/25 mM NH_4_Cl and PBS, permeabilized with 0.2% Triton X-100 (Sigma), washed twice with PBS and blocked with PBS/1% BSA (Fraction V, Sigma). Antibodies detecting viral infection were used as follows: mAB J2 (English & Scientific Consulting, Bt. Szirák, Hungary) to detect HRV and CV infections [21], and mAB HB65 detecting flu nucleoprotein (kind gift from J. Pavlovic, University of Zürich, Switzerland). For secondary reagents we used Alexa-fluor labelled antibodies (Invitrogen) together with 4′,6-diamidino-2-phenylindole (DAPI) to stain nuclei. Automated image acquisition was performed with an ImageXpress Micro (Molecular Devices) equipped with a CoolSNAP HQ 12bit grey-scale camera (Roper Scientific) and 10× SuperFluor Na 0.5, 20× SuperFluor Na 0.75, 40× SuperFLuor Na 0.95 (Nikon) objectives. Routinely, 9–20 images per well were acquired yielding an average of 5000–12000 cells analysed per well and data point. Image overlays where made using MetaXpress (Molecular Devices) and ImageJ (NIH Image, http://rsbweb.nih.gov/nih-image/). Images were analyzed using a custom written Matlab routine [21]. Dose response curve fittings were obtained with PRISM software (version 5.01, GraphPad Software Inc.) using standard Hill curve fittings. Infection indices were represented as a function of log_10_ inhibitor concentrations to determine the IC_50_ value for each drug, that is, the concentration of the drug provoking a response half way between the top response of the infection assay and the maximally inhibited response using variable slope model, representing a four-parameter dose-response curve.

### Full cycle infectivity assays

To determine the effect of compounds on the production of infectious virus, cells were treated as indicated and infected for 16 h. Progeny virus was recovered from the cells by three freeze/thaw cycles and debris separated from supernatants by centrifugation at 1000×g for 3 min. Titers from supernatants were determined with serial dilutions on HeLa-Ohio cells by staining cells with crystal violet and calculation of TCID_50_ values according to Spearman and Kärber [58]. A 3 h infection sample served as control for input virus carry-over.

### Compound assays, 19°C endosomal trapping experiments, pH rescue and capsid pre-incubation experiments

Compounds were dissolved in dimethyl sulfoxid (DMSO, cell culture grade, Sigma) and added to cells in dilutions indicated with respective concentrations of solvent alone as controls. For infection assays cells were pre-incubated with compound for 30 min or as indicated in infection medium. Virus was added to the cells at 37°C for 7 h in presence or absence of compounds, and cells were fixed and immune-stained for infection analyses. For endosomal trapping, viruses were added to cells in infection medium at 19°C and internalized for 1 to 3 h. The respective compounds diluted in infection medium were added in an equal volume 10 min before shifting the samples to 37°C for 7 h. Infection was quantified by immune-staining for dsRNA. For pH rescue, viruses were trapped at 19°C for 2 h in the presence of compound. The medium was replaced by buffers containing 150 mM NaCl and different pH with or without niclosamide, 25 mM HEPES-NaOH pH 7.0, 25 mM morpholine-ethane-sulfonic acid [MES] pH 6.5 or 6.0 or 25 mM sodium acetate pH 5.5 [24]. Cells were infected at 37°C for 1 h, and infection continued for another 6 h at 37°C in infection medium containing 1 µg/ml pleconaril to inactivate non-converted viruses. For capsid pre-incubation experiments with niclosamide the indicated viruses were either incubated in a small volume with niclosamide (5 µl) or in infection medium without compound. After 30 min incubation at 37°C virus was added to cells in 40 µl, followed either by addition of infection medium or niclosamide (5 µl) to reach the respective concentrations from the virus pre-treatment samples. After 7 h of infection cells were fixed and immune-stained for infection analyses.

### Intracellular acridine orange and lysotracker pH assays

For intracellular pH measurements, about 20,000 HeLa-Ohio cells were seeded into imaging compatible 96 well plates (Matrix, Thermo Scientific), washed the next day with PBS and incubated with compounds diluted in Hanks buffered salt solution (HBSS, Invitrogen) at 37°C for 30 min. An equal volume solution of AO (150 µg/ml, Life Technologies) and Hoechst 33342 (10 µg/ml, Sigma) or lysotracker DND99 (0.2 µM from a 1 mM stock in DMSO, Life Technologies) was added to the medium and incubated at 37°C for 15 min. Cells were washed twice with HBSS, and immediately imaged with ImageXpress Micro (Molecular Devices) equipped with a CoolSNAP HQ 12 bit grey-scale camera (Roper Scientific), a heating unit to keep the imaging stage at 37°C and a 20× SuperFluor/Na 0.75 objective (Nikon) and filter sets for AO (green: excitation 473/30 nm, emission 520/35 nm; red: excitation 479/28 nm, emission 607/36).

### Quantification of acridine orange, lysotracker and endosomal stainings

Quantification was performed using the open source image analysis framework CellProfiler Version 2 (www.cellprofiler.org). The raw fluorescence intensities were quantified in the perinuclear area. Raw fluorescence intensities were from lysotracker DND99. The ratios of green to red fluorescence (AO) or fluorescence intensities of compartments stained for early endosomal antigen 1 (EEA1, rabbit polyclonal antibody, Novus Biologicals, Cat# NB300-502) or LAMP1 (mouse H4A3 antibody, Santa Cruz Biotechnology) were measured relative to untreated cells.

### Acridine orange measurements in clathrin-coated vesicles

CCV suspended in assay buffer (10 mM HEPES, pH7, 100 mM KCl, 1 mM EDTA) were incubated with 1 mM ATP, 2 µM AO (Molecular Probes). The ATPase reaction was initiated by adding MgCl_2_ to 1 mM and fluorescence was determined at 492/540 nm in a plate reader (Tecan, Männedorf, Switzerland) at room temperature after 10 min. BafA1 and samples without MgCl_2_ were used as controls.

### v-ATPase inhibition assay

Cow brain CCV were prepared as described [59], diluted in assay buffer (200 mM sucrose, 50 mM KCl, 10 mM HEPES pH7, 1 mM EDTA, 5 µM nigericin, 10 µg/ml oligomycin, 1 mM vanadate) and incubated with the respective compounds at 4°C for 30 min. ATP (10 µl 2 mM ATP and 40 mM MgCl_2_) were added and the samples incubated at 37°C for 15 min. Production of inorganic phosphate (P_i_) was measured with the Sensolyte MG phosphate assay kit according to the manufacturers protocols (AnaSpec).

### Compound toxicity measured by resazurin fluorescence

Cell toxicity was assessed by the resazurin fluorometric method [60]. In brief, sterile filtered resazurin (1 mg/ml stock solution in PBS, Sigma) was stored protected from light at 4°C, and added to cells to a final concentration of 0.16 µg/ml together with the compounds of interest and incubated at 37°C in a 5% CO_2_ incubator as indicated. The conversion of resazurin to a fluorescent product was measured using a plate reader (TECAN Infinite 200) at excitation 550/9 nm and emission 590/20 nm wavelength at constant gain over time at 37°C.

### Liposome dextran-FITC de-quenching

Liposomes from soy phosphatidyl-choline were prepared in 100 mM acetate pH 5.15 containing 1 mM 4.4 kDa FITC-dextran using standard extrusion protocols yielding vesicles of an average size of 200 nm measured using a particle size analyzer (Nicomp 370, Nicomp, Port Richey, FL, USA). Non-encapsulated FITC-dextran was removed by size exclusion chromatography in a G25 column (Pharmacia) followed by extensive dialysis against 100 mM acetate pH 5.15 in a Floatalyzer with 50 kDa cut-off (Spectrumlabs). To measure fluorescence de-quenching FITC-dextran containing liposomes were diluted in PBS at 20–60 µg/ml, incubated with the respective compounds at room temperature and analysed for fluorescence using a plate reader (TECAN Safire II) with fluorescence TOP measurement settings at excitation of 483/10 nm and emission of 525/15 nm wavelength within 15 min. Control measurements of samples containing PBS, Triton X-100 or the compounds alone were carried out to measure background, maximal readout of the assay and auto-fluorescence of compounds. The data were normalized to the PBS control and background fluorescence was subtracted.

### Synergy analyses

Cells were pre-treated with compounds alone or in combination, infected as indicated and analyzed using immune-staining and automated microscopy as described above. MacSynergy II [61] was used to calculate synergy interaction indices. Briefly, theoretical additive interactions were calculated from the dose-response curves of each individual drug. The calculated additive surface was then subtracted from the experimental surface to obtain a synergy surface representing percentage inhibition above the calculated additive value. Any peak above the 0% plane suggests synergy. Likewise, any peak below the 0% plane is indicative of antagonism.

## Supporting Information

Text S1 **This file contains supporting figures S1 to S8 and references. Figure S1.** Niclosamide is a dose-dependent low micro-molar inhibitor of HRV in HeLa cells. **Figure S2.** Pre-incubating HRV or CVB3 with niclosamide has no effects on infectivity. **Figure S3.** Time course of bafilomycin A1 addition to cells infected with HRV1A, 2, 14, 16 and the less pH dependent CVB4, and the pH independent CVB3. **Figure S4.** No decrease in antiviral efficacy of niclosamide upon HRV1A passage at low drug concentrations. **Figure S5.** Niclosamide is an early and reversible inhibitor of HRV1A, 2, 14 and 16 infections.**Figure S6.** Niclosamide affects acidic compartments similar to BafA1 or ammonium chloride but has no effect on the v-ATPase activity in CCV preparations. **Figure S7.** Co-tracking of antiviral efficacy with endosomal pH neutralization of niclosamide-related compounds. **Figure S8.** Synergistic inhibition of HRV1A infection by low concentrations of niclosamide and BafA1.
(PDF)
