# Supplementary material for: Niclosamide Is a Proton Carrier and Targets Acidic Endosomes with Broad Antiviral Effects
Source: PLoS Pathog. 2012 Oct 25;8(10):e1002976. doi: 10.1371/journal.ppat.1002976 (PMC3486884; doi:10.1371/journal.ppat.1002976)
Supplement: Text S1 — This file contains supporting figures S1 to S8 and references. Figure S1. Niclosamide is a dose-dependent low micro-molar inhibitor of HRV in HeLa cells. Figure S2. Pre-incubating HRV or CVB3 with niclosamide has no effects on infectivity. Figure S3. Time course of bafilomycin A1 addition to cells infected with HRV1A, 2, 14, 16 and the less pH dependent CVB4, and the pH independent CVB3. Figure S4. No decrease in antiviral efficacy of niclosamide upon HRV1A passage at low drug concentrations. Figure S5. Niclosamide is an early and reversible inhibitor of HRV1A, 2, 14 and 16 infections.Figure S6. Niclosamide affects acidic compartments similar to BafA1 or ammonium chloride but has no effect on the v-ATPase activity in CCV preparations. Figure S7. Co-tracking of antiviral efficacy with endosomal pH neutralization of niclosamide-related compounds. Figure S8. Synergistic inhibition of HRV1A infection by low concentrations of niclosamide and BafA1. (PDF) [file ppat.1002976.s001.pdf]

Supporting Text S1

Figure S1

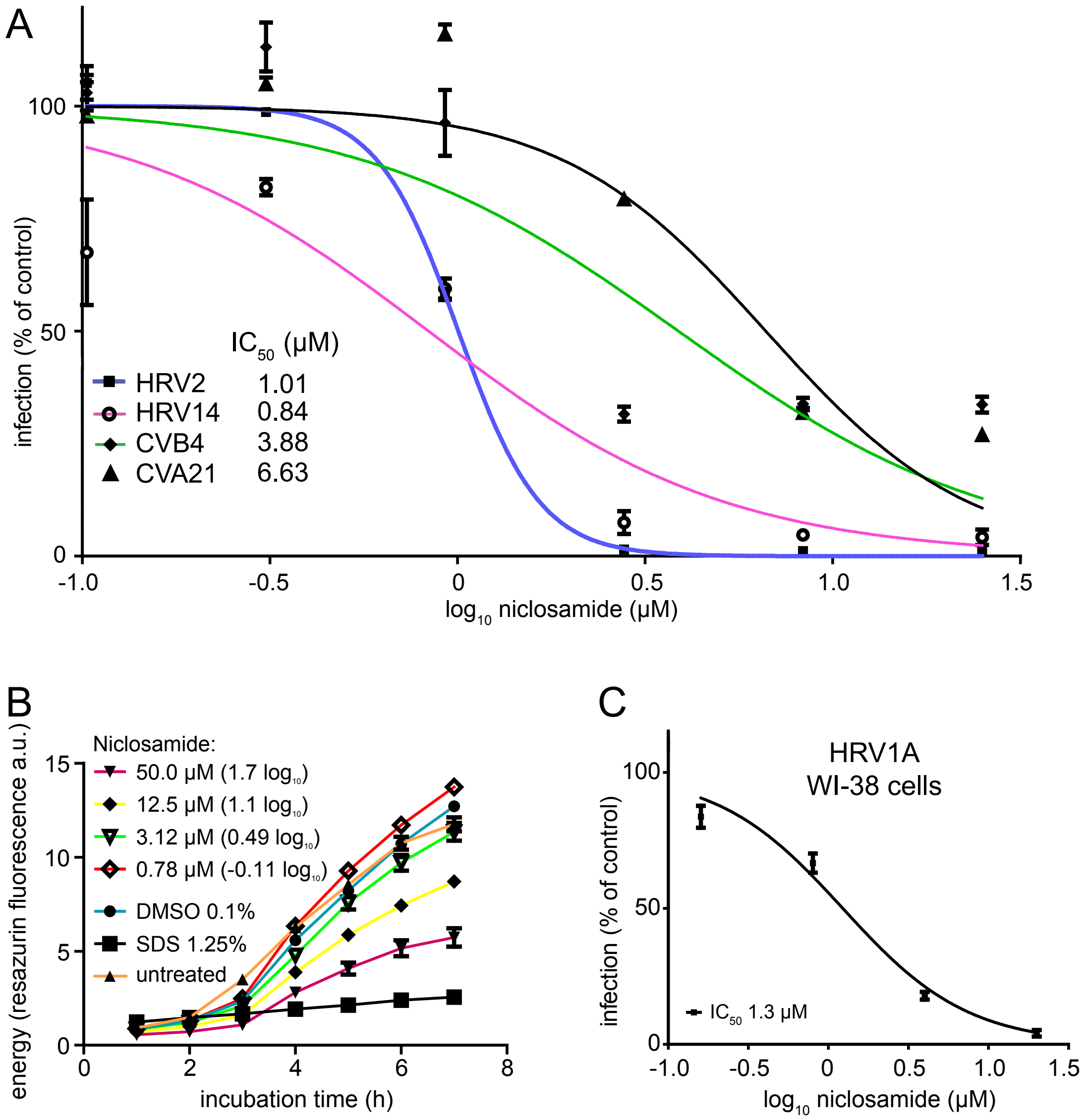

Figure S1: Niclosamide is a dose-dependent low micro-molar inhibitor of HRV in HeLa cells.

A) HeLa cervical carcinoma cells pre-incubated with niclosamide are protected from infection by HRV2, 14 (for HRV1A & 16, see Figure 1) at concentrations in the range of 1 µM, and less protected from CVB4 and A21 (for CVB3, see Figure 1). Infection was measured by the formation of dsRNA replication centers and quantified using automated microscopy and single cell image analysis [1]. For all experiments the average infection index and SEM from two independent experiments are shown. Data were normalized to DMSO treated control samples (100%).

B) Time course of metabolic activity in HeLa cells determined by resazurin conversion and change in fluorescence in the presence of increasing concentrations of niclosamide, SDS or DMSO. Mean and SEM values are shown (n=16).

C) Niclosamide protects WI-38 human lung fibroblasts from infection with HRV1A. Infection was measured as described in a). Mean and SEM values shown (n=4).

Figure S2

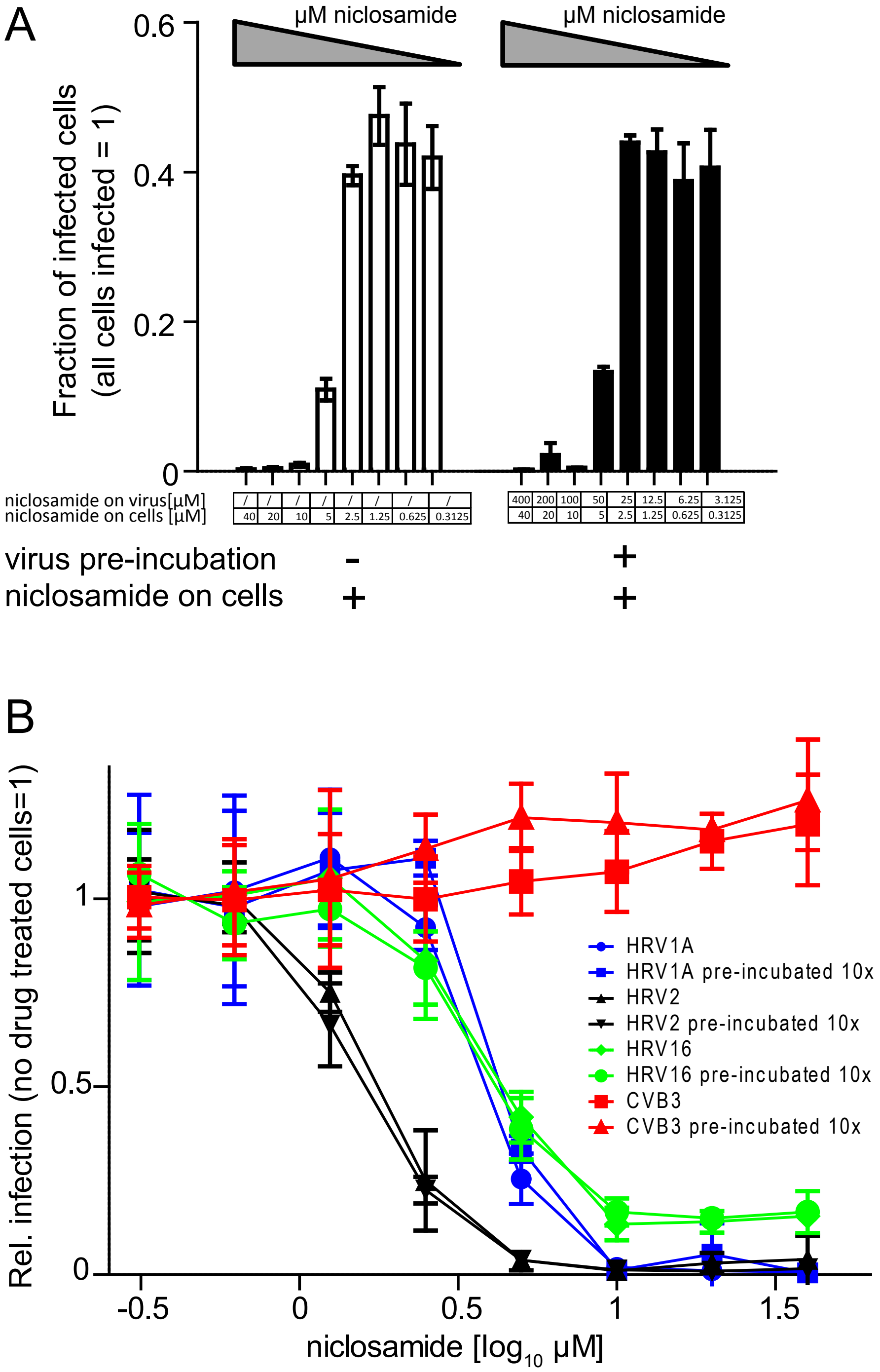
A
µM niclosamide
µM niclosamide
0.6
0.4
0.2
0
Fraction of infected cells
(all cells infected = 1)
niclosamide on virus[µM]
niclosamide on cells [µM]
/ / / / / / / /
40 20 10 5 2.5 1.25 0.625 0.3125
400 200 100 50 25 12.5 6.25 3.125
40 20 10 5 2.5 1.25 0.625 0.3125
virus pre-incubation - +
niclosamide on cells + +
B
Rel. infection (no drug treated cells=1)
1
0.5
0
-0.5
0
0.5
1
1.5
niclosamide [log10 µM]
HRV1A
HRV1A pre-incubated 10x
HRV2
HRV2 pre-incubated 10x
HRV16
HRV16 pre-incubated 10x
CVB3
CVB3 pre-incubated 10x

Figure S2: Pre-incubating HRV or CVB3 with niclosamide has no effects on infectivity.

A) HRV1A was pre-treated without (left panel) or with niclosamide (right panel) in infection medium at 37°C for 30 min, diluted 10-fold into infection medium, added to cells for 7 h followed by infection analyses as described in materials and methods. Cells inoculated with virus, which was not pre-treated with niclosamide were treated with drug concentrations corresponding to the concentrations from the pre-treated viruses. Note that there was no difference in the infection profile from viruses pre-treated or not with niclosamide.

B) Infection profiles from HRV1A, 2, 16 and CVB3 pre-treated or not with different concentrations of niclosamide. The experiment was carried out as described in panel a) with the addition that data were normalized to the respective untreated infection control in order to allow easier comparison between different viruses. The data show closely overlapping inhibition curves independent of whether the viruses were pre-treated with the drug or not.

Figure S3

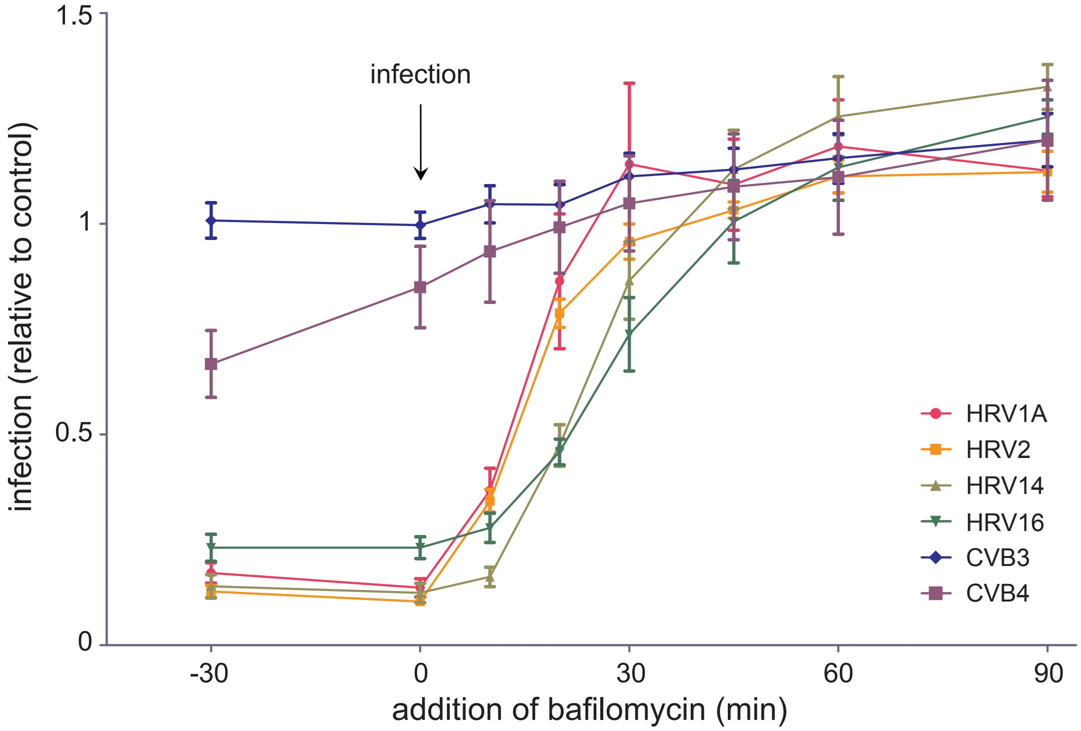
1.5
1
0.5
0
infection (relative to control)
infection
HRV1A
HRV2
HRV14
HRV16
CVB3
CVB4
-30
0
30
60
90
addition of bafilomycin (min)

Figure S3: Time course of bafilomycin A1 addition to cells infected with HRV1A, 2, 14, 16 and the less pH dependent CVB4, and the pH independent CVB3.

HeLa cells were either pre-incubated for 30 min with bafilomycin A1 (BafA1, 50nM), or drug was added at the time of infection (0 min) or time points post infection with HRV1A, 2, 14, 16 or CVB3, B4. Infection was at 37°C for 7 h and scoring of infected cells as described in the main text.

Figure S4

A Passage of HRV1A in niclosamide does not raise drug resistance

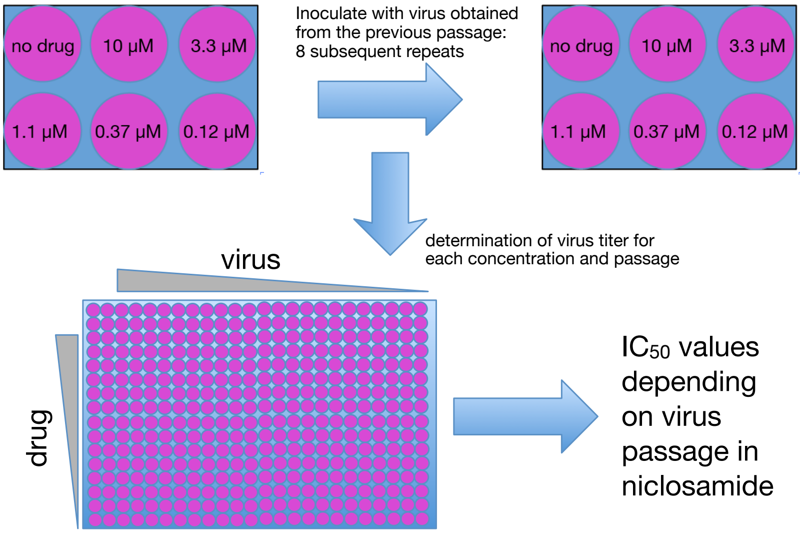

Viruses were passaged eight times in the presence of different concentrations of niclosamide. Each of the 48 resulting virus preparations were titrated in the presence of niclosamide and IC_{50} values were obtained.

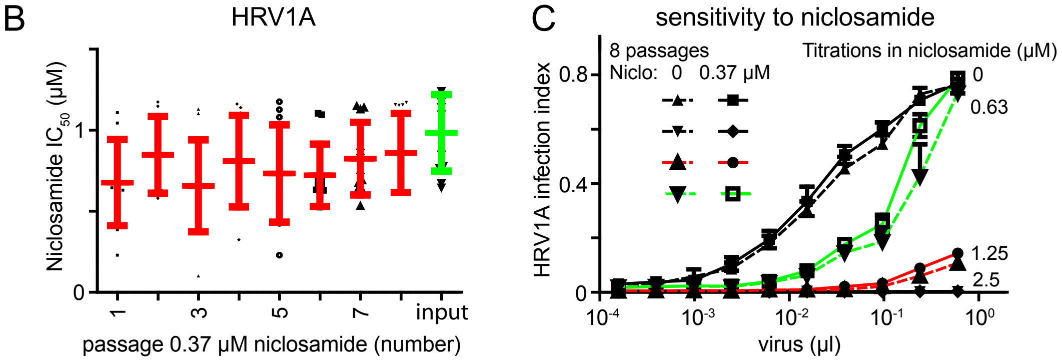

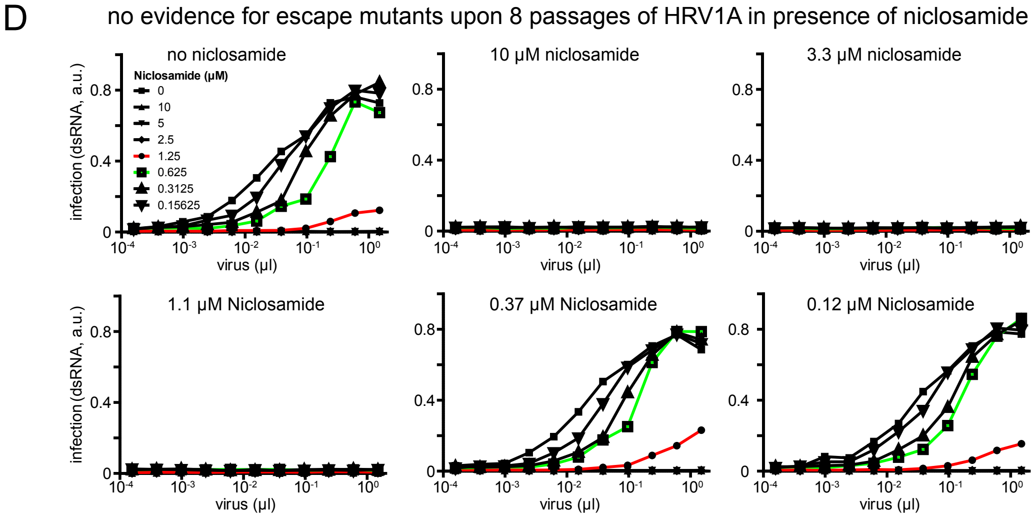

Figure S4: No decrease in antiviral efficacy of niclosamide upon HRV1A passage at low drug concentrations.

A) Schematic drawing depicting the passage and titration of HRV1A with different concentrations of niclosamide.

B) HRV1A was passaged eight times for 24 h in HeLa cells in the presence of 0.37 μM niclosamide and titrated against increasing concentrations of niclosamide up to 10 μM. The mean and SEM of IC_{50} values of 11 virus concentrations are plotted (red), the input virus in green.

C) Sensitivity to niclosamide of HRV1A passaged either eight times in niclosamide (0.37 μM) or untreated virus. Shown are mean and SEM from 4 experiments.

D) No evidence for HRV1A escape mutants upon virus passage in niclosamide containing media. Viruses collected from infected cells by freeze/thaw and passaged for eight times 24 h at different concentrations of niclosamide showed no shift of sensitivity against the drug. Infection was measured by the formation of dsRNA replication centers and quantified using automated microscopy and single cell image analysis. For all experiments the average and SEM values from four independent experiments are shown.

Figure S5

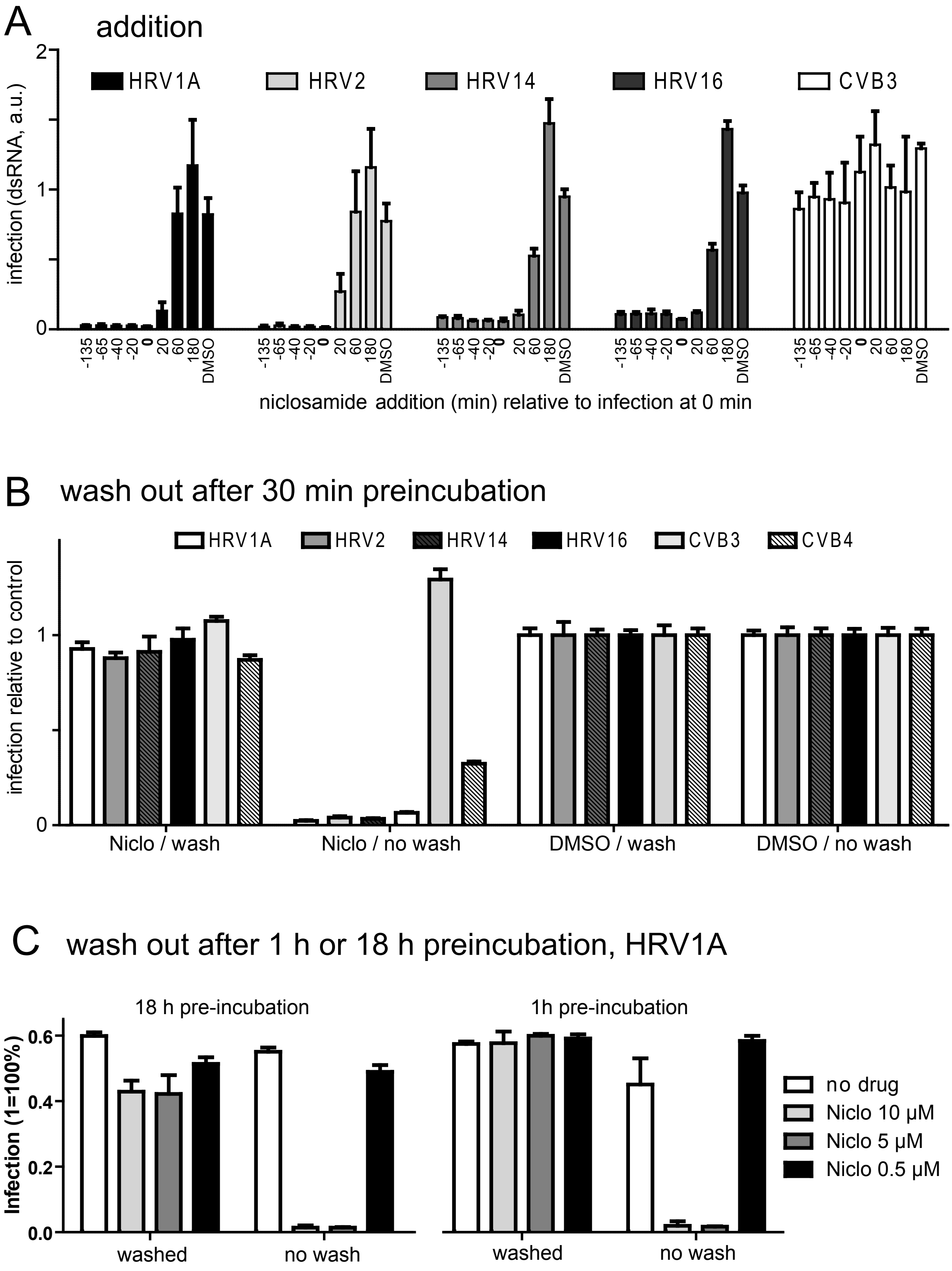
A
addition
HRV1A
HRV2
HRV14
HRV16
CVB3
infection (dsRNA, a.u.)
2
1
0
-135
-65
-40
-20
0
20
60
180
DMSO
niclosamide addition (min) relative to infection at 0 min
B
wash out after 30 min preincubation
HRV1A
HRV2
HRV14
HRV16
CVB3
CVB4
infection relative to control
1
0
Niclo / wash
Niclo / no wash
DMSO / wash
DMSO / no wash
C
wash out after 1 h or 18 h preincubation, HRV1A
18 h pre-incubation
1h pre-incubation
Infection (1=100%)
0.6
0.4
0.2
0.0
washed
no wash
no drug
Niclo 10 μM
Niclo 5 μM
Niclo 0.5 μM

Figure S5: Niclosamide is an early and reversible inhibitor of HRV1A, 2, 14 and 16 infections.

A) Quantification of the impact of niclosamide addition before and during infection (time point 0) on infection levels of HRV1A, 2, 14 and 16 and CVB3 quantified by automated microscopy and single cell quantification. Mean and SEM values shown (n=4).

B) Impact of drug washout on the efficacy of niclosamide against HRV1A, 2, 14, 16, CVB3 and B4 infections of HeLa cells. Cells pre-incubated with 10 μM of niclosamide for 30 min were either washed three times with PBS or virus was added in the presence of the drug. Infections were quantified as described in panel (A), and means and SEM values from four independent experiments are shown.

C) Analogous to panel (B) cells where pre-treated with niclosamide, washed and infected with HRV1A. In addition to the treatment shown in panel (b), 3 different concentrations of niclosamide were applied and pre-incubation times were 1 h or 18 h.

Figure S6

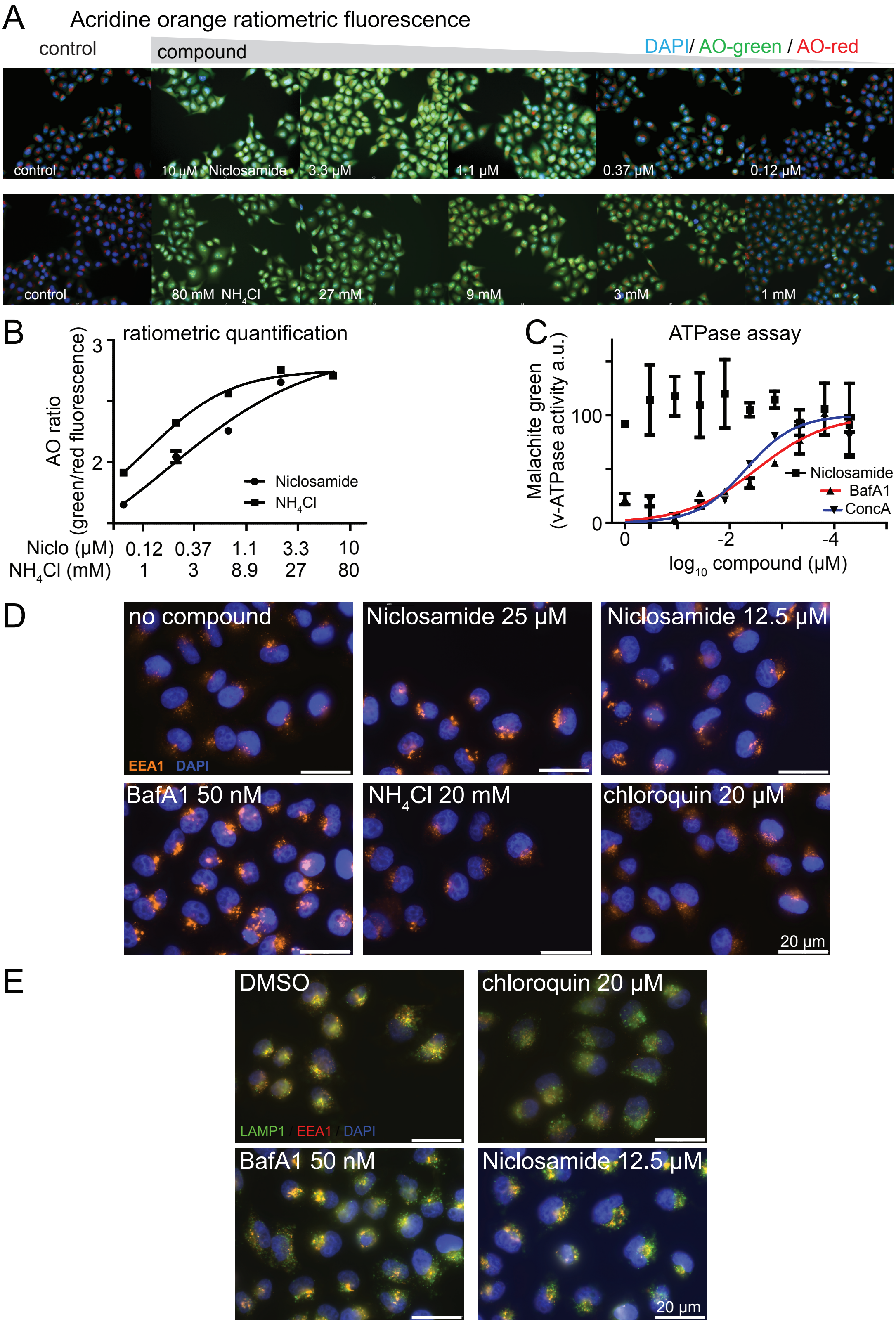
A
Acridine orange ratiometric fluorescence
control
compound
DAPI/ AO-green / AO-red
control
10 µM Niclosamide
3.3 µM
1.1 µM
0.37 µM
0.12 µM
control
80 mM NH4Cl
27 mM
9 mM
3 mM
1 mM
B
ratiometric quantification
AO ratio (green/red fluorescence)
Niclosamide
NH4Cl
Niclo (µM) 0.12 0.37 1.1 3.3 10
NH4Cl (mM) 1 3 8.9 27 80
C
ATPase assay
Malachite green (v-ATPase activity a.u.)
Niclosamide
BafA1
ConcA
log10 compound (µM)
D
no compound
Niclosamide 25 µM
Niclosamide 12.5 µM
EEA1 DAPI
BafA1 50 nM
NH4Cl 20 mM
chloroquin 20 µM
20 µm
E
DMSO
chloroquin 20 µM
LAMP1 EEA1 DAPI
BafA1 50 nM
Niclosamide 12.5 µM
20 µm

Figure S6: Niclosamide affects acidic compartments similar to BafA1 or ammonium chloride but has no effect on the v-ATPase activity in CCV preparations.

A) Niclosamide induces the neutralization of intracellular vesicles as measured by ratiometric live cell imaging of AO green and red fluorescence with DAPI staining for nuclei (blue).
B) Quantification of AO green/red fluorescence ratio of HeLa cells treated with niclosamide or NH_4Cl. Perinuclear fluorescence intensity was quantified by high throughput automated live cell microscopy and single cell image analysis. Mean and SEM values shown (n=4).
C) Niclosamide does not inhibit v-ATPase activity in bovine brain CCV preparations measured in a plate reader format assay using malachite green detection of P_i.
D) Impact of niclosamide, BafA1, NH_4Cl or chloroquine on the distribution of early endosomal structures stained by EEA1 (red) in HeLa cells, including DAPI stained nuclei (blue).
E) Impact of niclosamide, BafA1 or chloroquine on the distribution of early endosomal structures stained by EEA1 (red) and late endosomes stained by LAMP1 (green) in HeLa cells.

Figure S7

A

anti-viral efficacy

		DMSO	25.0 [µM]	8.3 [µM]	2.8 [µM]	0.9 [µM]	0.3 [µM]	0.1 [µM]	no virus	IC50
HRV1A	TVB-1326	0.95	0.02	0.17	0.62	0.84	0.91	0.93	0.01	3.36
	TVB-1329	1.02	0.05	0.59	1.02	1.10	1.04	1.00	0.01	9.34
	TVB-1332	1.00	0.02	0.04	0.61	0.97	1.01	0.99	0.01	3.19
	TVB-1334	0.96	0.02	0.11	0.70	0.88	0.95	0.94	0.01	3.79
	TVB-1338	1.01	0.95	1.11	1.12	1.07	0.96	1.02	0.01	
	Niclosamide	1.05	0.02	0.01	0.02	0.62	1.01	1.10	0.01	1.04
HRV2	TVB-1326	1.03	0.01	0.15	0.72	0.86	0.96	1.05	0.01	3.98
	TVB-1329	1.02	0.02	0.36	0.68	0.98	1.10	1.03	0.01	5.07
	TVB-1332	1.04	0.01	0.01	0.38	0.95	0.97	0.94	0.01	2.36
	TVB-1334	0.95	0.01	0.03	0.72	0.98	1.01	1.05	0.01	3.53
	TVB-1338	0.94	0.76	1.12	1.13	1.12	1.11	1.05	0.01	
	Niclosamide	1.01	0.01	0.01	0.02	0.58	0.99	1.05	0.01	1.01
HRV14	TVB-1326	1.05	0.07	0.46	0.81	0.88	0.90	0.87	0.02	6.87
	TVB-1329	0.91	0.10	0.58	0.86	0.97	0.87	0.86	0.01	9.30
	TVB-1332	0.96	0.05	0.09	0.47	0.82	0.96	0.91	0.02	2.47
	TVB-1334	0.93	0.09	0.17	0.90	0.74	0.85	1.06	0.04	5.19
	TVB-1338	1.13	0.62	1.13	1.17	1.20	1.12	1.07	0.02	
	Niclosamide	1.04	0.04	0.05	0.07	0.59	0.82	0.68	0.01	0.84
HRV16	TVB-1326	1.00	0.10	0.68	0.96	0.96	1.00	0.99	0.01	11.11
	TVB-1329	1.02	0.14	0.99	1.26	1.04	0.98	0.84	0.01	
	TVB-1332	0.98	0.07	0.14	0.66	0.94	0.95	0.98	0.01	3.74
	TVB-1334	1.05	0.10	0.34	0.89	1.07	1.04	0.95	0.01	6.50
	TVB-1338	0.98	0.67	1.02	1.13	1.15	1.11	1.09	0.01	~ 26.87
	Niclosamide	0.98	0.07	0.08	0.13	0.74	0.92	0.84	0.01	1.38
CVA21	TVB-1326	0.97	0.59	1.10	0.99	1.00	0.98	0.87	0.01	~ 25.76
	TVB-1329	1.00	0.68	1.29	1.13	1.08	0.97	0.86	0.01	~ 26.63
	TVB-1332	1.00	0.20	0.81	1.02	0.93	0.95	0.91	0.01	14.68
	TVB-1334	1.07	0.30	1.18	1.21	1.05	0.97	0.98	0.01	~ 23.46
	TVB-1338	0.99	0.81	1.06	1.05	1.03	1.05	1.02	0.01	
	Niclosamide	0.98	0.27	0.32	0.79	1.16	1.05	0.98	0.01	6.63
CVB3	TVB-1326	1.00	1.27	1.00	1.06	1.11	1.06	1.05	0.01	
	TVB-1329	1.00	1.01	1.07	1.25	1.19	1.06	0.97	0.00	
	TVB-1332	1.06	1.15	1.05	1.08	1.07	1.11	1.01	0.01	
	TVB-1334	1.03	1.27	1.17	1.08	1.14	1.12	1.09	0.01	
	TVB-1338	0.99	0.94	0.94	1.05	1.09	1.10	0.97	0.01	
	Niclosamide	0.92	1.11	1.10	0.91	0.83	0.91	0.93	0.01	
CVB4	TVB-1326	1.05	0.29	0.71	1.06	1.20	1.22	1.13	0.01	15.01
	TVB-1329	0.98	0.46	1.33	1.62	1.51	1.28	1.12	0.01	
	TVB-1332	1.02	0.33	0.34	0.86	1.25	1.16	1.10	0.01	8.01
	TVB-1334	1.05	0.56	0.49	1.23	1.26	1.14	1.11	0.01	21.21
	TVB-1338	0.94	0.51	1.02	1.15	1.06	1.02	1.00	0.01	
	Niclosamide	0.96	0.34	0.34	0.32	0.96	1.13	1.03	0.01	3.88
PR8	TVB-1326	1.00	0.01	0.68	0.78	0.90	0.94	1.02	0.00	10.24
	TVB-1329	1.00	0.04	0.69	0.99	1.04	0.98	0.99	0.00	10.34
	TVB-1332	1.00	0.00	0.02	0.41	1.04	1.06	1.04	0.00	~ 2.70
	TVB-1334	1.00	0.00	0.41	0.82	0.83	0.90	0.99	0.00	6.24
	TVB-1338	1.00	0.40	0.78	0.82	0.78	0.81	0.81	0.00	42.91
	Niclosamide	1.00	0.00	0.00	0.01	0.45	0.89	0.96	0.00	0.83
	relative infection	1.40	1.20	1.00	0.80	0.60	0.40	0.20	0.00	

C

low pH neutralization (lysotracker assay)

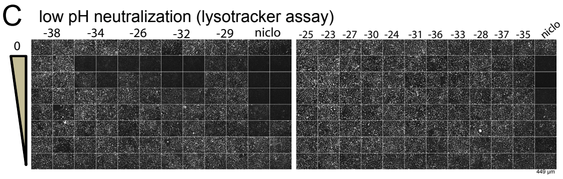

B

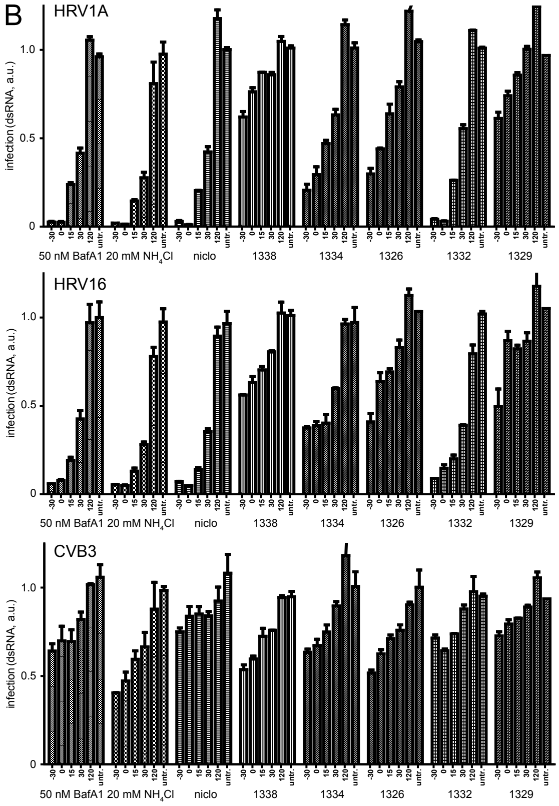

Figure S7: Co-tracking of antiviral efficacy with endosomal pH neutralization of niclosamide-related compounds.

A) Dose-dependent antiviral efficacy of niclosamide and five structurally related compounds TVB-1326, 1329, 1332, 1334 and 1338 was tested against a panel of seven picornaviruses (HRV1A, 2, 14, 16, CVA21, B3 and B4) and the influenza strain PR8. Infection was measured by the formation of dsRNA replication centers, or nucleoprotein expression for influenza virus. Quantification was done by automated microscopy and single cell image analysis, and mean infection values are shown from two independent experiments, normalized to DMSO treated control cells. IC_{50} values (µM) representing half maximal inhibition concentrations of the respective compounds are indicated on the right side. Color code indicates reduction (red) or increase (green) of infection relative to DMSO control (1, black).

B) Time course of addition of pH neutralizing agents BafA1, NH_4Cl, niclosamide or TVBs (10 µM). HeLa cells were either pre-incubated with compounds, or compounds were added at the indicated time points post infection. Graphs show the mean infection and SEM values (n=2).

C) Impact of niclosamide and sixteen structurally related compounds on the accumulation of the acidotropic dye lysotracker DND99 in HeLa cells. Overview montages acquired by high throughput live cell microscopy in 96-well plates are shown. Each image represents a field of view in one well measuring 449 µm by 335.5 µm.

Figure S8

A

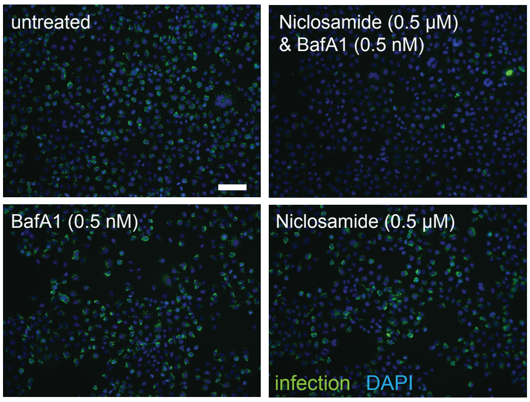

B

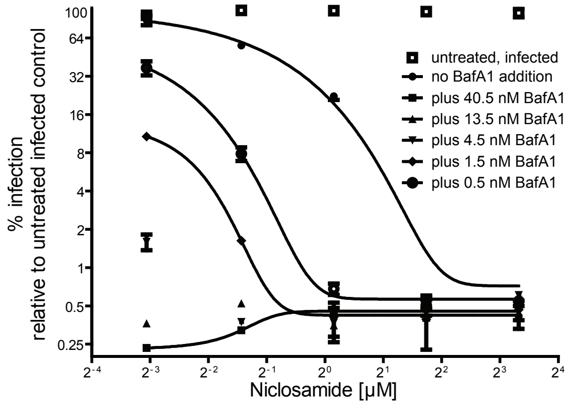

C

Synergy determination using MacSynergy II

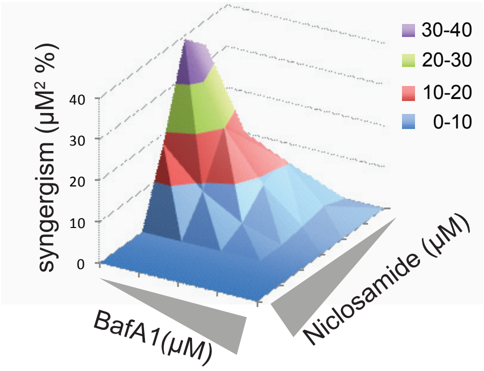

SYNERGY PLOT (99.9%)	
Bonferroni Adj.	98%
**SYNERGY**	**235.5**
log volume	53.6
ANTAGONISM	-1.4
log volume	-0.32

Figure S8: Synergistic inhibition of HRV1A infection by low concentrations of niclosamide and BafA1.

A) HeLa cells were incubated with serial dilutions of niclosamide (10 µM and 3-fold serial dilutions thereof) and BafA1 (40.5 nM and 3-fold serial dilutions thereof) and combinations thereof followed by infection with HRV1A for 16 h, and staining for infected cells with mAB J2 detecting dsRNA replication centers. Representative images of cells treated with BafA1 and niclosamide and combinations thereof are shown. Infected cells are shown in green and nuclei stained with DAPI in blue. Scale bar 100 µm.

B) Inhibition of HRV1A infection by serial dilutions of niclosamide combined with serial dilutions of BafA1. Infection was scored by immune-staining for dsRNA replication centers, followed by automated imaging and image analysis.

C) Representation of synergy values calculated with MacSynergy II, as developed by [2]. Synergy levels are plotted as a function of BafA1 (µM) and niclosamide concentrations (µM) showing greatest level of synergy at concentrations below 0.5 µM for niclosamide and 0.5 nM for BafA1. Results were derived from n=2 experiments.

Supporting references

1. Jurgeit A, Moese S, Roulin P, Dorsch A, Loetzerich M, et al. (2010) An RNA replication-center assay for high content image-based quantifications of human rhinovirus and coxsackievirus infections. Virology Journal 7: 264.
2. Prichard MN, Shipman C, Jr. (1990) A three-dimensional model to analyze drug-drug interactions. Antiviral Res 14: 181-205.
